# Remote diffuse multi-coronary artery spasm following pulmonary vein and posterior wall isolation, using a focal monopolar pulsed field ablation catheter

**DOI:** 10.1016/j.hrcr.2025.08.003

**Published:** 2025-08-06

**Authors:** James Mannion, Colin Gorry, Brendan Foley, Stephen Tuohy

**Affiliations:** 1 Cardiology Department, Beacon Hospital, Sandyford, Dublin, Ireland; 2 Cardiology Department, Mater Misericordiae University Hospital, Eccles Street, Dublin, Ireland

**Keywords:** Coronary artery spasm, Pulsed field ablation, Kounis syndrome, Protamine, Sugammadex, Cardiac arrest

**Key Teaching Points**

• Late, remote coronary artery spasm may occur after pulsed field ablation for atrial fibrillation.
• This is the first case to demonstrate the spasm of all coronary arteries in this setting.
• This was a high-risk complication resulting in cardiac arrest.
• The 450 mcg intracoronary nitrate was insufficient to resolve the spasm in all 3 vessels; therefore, limited by hypotension, a drug-eluting balloon was required.

## Introduction

Ablation with pulsed field (PF) energy generates irreversible electroporation in cardiac myocytes, inducing apoptosis and death, with resultant scar and electrophysiological block. The uptake of PF energy in catheter ablation continues to rapidly increase worldwide. Although PF was initially suspected to have fewer complications due to being more cardio-selective than thermal energies, reports of clinical coronary artery spasm have also increased in number, with this phenomenon widely accepted as a class effect.^1^, ^2^, ^3^ Spasm generally occurs acutely when PF energy is deployed in close proximity to the coronary arteries, such as during cavo-tricuspid isthmus or mitral isthmus line ablation. Vascular smooth muscle cells can be effected by multiple mechanisms, such as direct electrical stimulation to contract or disruption of the nitric oxide pathway.^4^^,^^5^ If blood pressure will allow, prophylaxis or treatment is generally with intravenous nitrates.^3^

PF technologies are evolving rapidly, with different delivery modalities and configurations, which may result in different coronary artery effects. There have been case reports of focal coronary artery spasm occurring remotely from the PF energy application in space or time.^6^, ^7^, ^8^ Herein, we describe the case and discuss mechanisms of diffuse remote spasm of multiple coronary arteries after pulmonary vein isolation (PVI) and posterior wall isolation (PWI) with a wide footprint monopolar lattice-tipped catheter resulting in cardiac arrest.

## Case report

A 64-year-old man with recurrent symptomatic atrial fibrillation despite anti-arrhythmic drug therapy was brought to the electrophysiology lab for repeat PVI. He had his first PVI 5 months before, involving wide antral circumferential ablation (WACA) of the veins with radiofrequency (RF) energy. He had no other significant past medical history, and had a normal coronary angiogram 6 years prior. His medications included bisoprolol 5 mg once daily, flecainide 50 mg twice daily (BD), and apixaban 5 mg BD.

**Figure 1:**
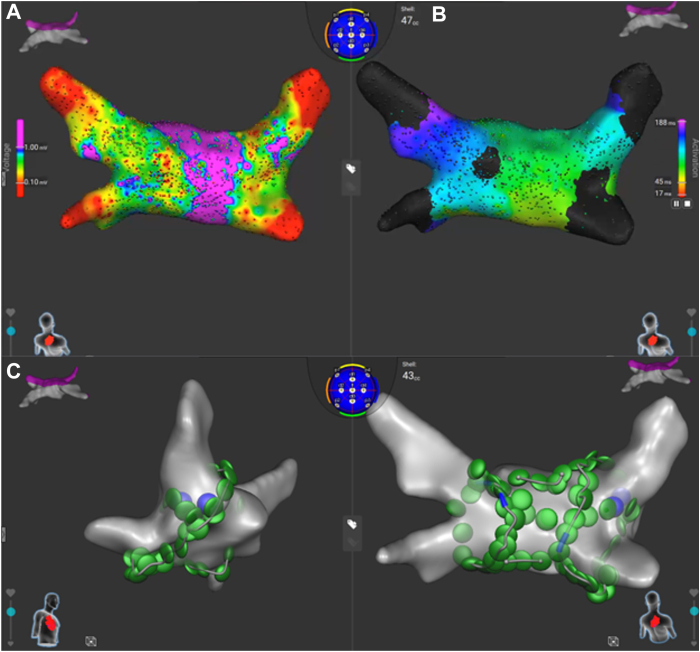
Ablation maps. **A:** Poster-anterior (PA) view of the left atrium (LA), with evidence of low voltage areas on the posterior wall. **B**: Local activation time map of the posterior wall. **C**: Lateral and PA views of the left atrium, demonstrating ablation tags of the wide antral circumferential ablation with posterior wall isolation.

The PVI was performed under general anesthesia via a standard approach. The right femoral vein was accessed via ultrasound guidance. Heparin was administered to maintain an activated clotting time of 350–400 seconds. A multi-electrode catheter was placed in the coronary sinus (DECANAV^TM^, Biosense Webster). Transesophageal echo and fluoroscopy were used for the trans-septal puncture. A steerable sheath (Vizigo^TM^, Biosense Webster) was introduced to the left atrium and a dual energy lattice-tipped mapping and monopolar ablation catheter (Sphere-9^TM^, Affera mapping and ablation system, Medtronic Inc) was advanced. A high-density left atrial electro-anatomical map was generated. Pulmonary venous reconnections were noted on both the left- and right-sided veins. Additionally, areas of abnormal voltage and slow conduction was identified on the posterior wall (Figure 1A and 1B). A repeat WACA PVI with a box PWI was performed (Figure 1C).

The procedure was uneventful, and acute markers of procedural success, including entrance and exit block to/from the veins and posterior wall were achieved. A total of 68 applications of PF were administered, which comprise 102,000 pulses. Protamine was given to reverse heparinization, femoral venous sheaths were removed from the body, and hemostasis was achieved with manual pressure. The patient remained hemodynamically stable, with no evidence of ST-segment changes, bradycardia, or heart block during the case.

Medications used at induction and early in the procedure included midazolam 3 mg, fentanyl 100 mcg, propofol 200 mg, rocuronium 100 mg, heparin as per activated clotting time, dexamethasone 16 mg, paracetamol 1 g, dexketoprofen 50 mg, ondansetron 4 mg, and phenylephrine 60 mcg. At the end of the procedure, protamine 50 mg, doxapram 80 mcg, sugammadex 400 mg, and flumazenil 400 mcg were administered for extubation.

**Figure 2:**
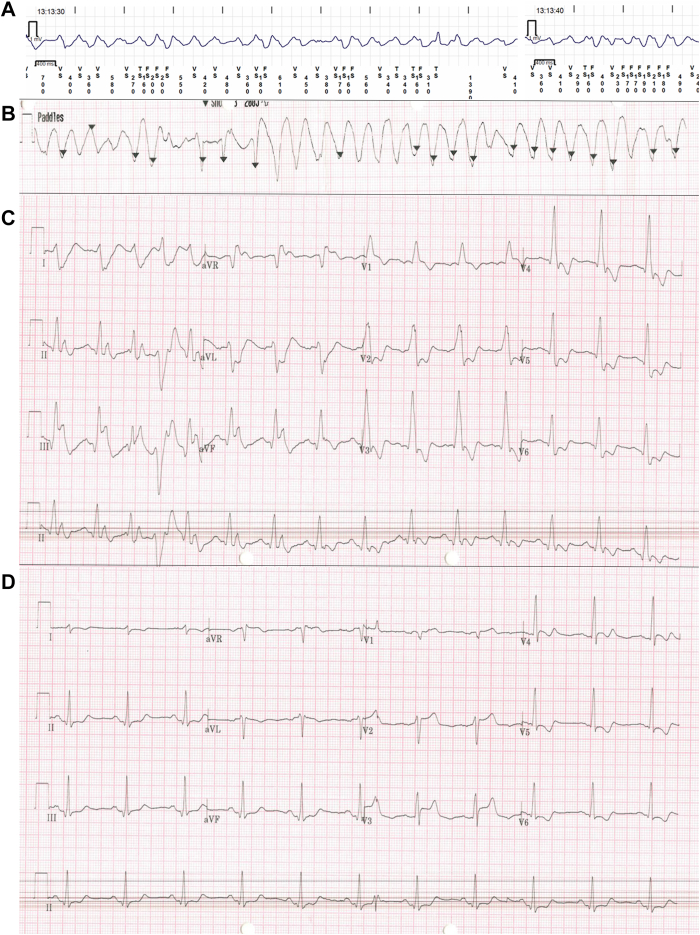
Polymorphic ventricular tachycardia on loop recorder and pads, followed by 12-lead ECGs. **A:** Polymorphic ventricular tachycardia (VT) retrieved from the patient loop recorder, identifying the onset time. **B:** Polymorphic VT on defibrillator pads trace, briefly self-terminating before restarting resulting in shock. **C: A** 12-lead ECG post return of spontaneous circulation, with an interventricular conduction delay and ST-segment elevation in the inferior leads with reciprocal ST depression in high lateral and anterior leads. **D:** ECG was repeated 3 minutes after **B**, demonstrating different territory ST segment changes, now in the anterior leads. ECG = electrocardiogram.

Very shortly after extubation, the patient lost cardiac output with the cardiac monitor and his loop recorder picked up polymorphic ventricular tachycardia (Figure 2A and 2B). This required 3 shocks of 200 joules to achieve return of spontaneous circulation (ROSC). The immediate post-ROSC electrocardiogram (ECG) showed a markedly prolonged QRS (180 ms), with right bundle branch block and ST-segment elevation in the inferior leads with reciprocal changes (Figure 2C). Three minutes later an ECG was repeated, and the right bundle branch block had improved; however, now there was ST-segment elevation in a different territory, the antero-septal leads V2–V3. There were reciprocal changes in V5 and V6, suggesting dynamic critical stenoses in multiple coronary arteries (Figure 2D).

**Figure 3:**
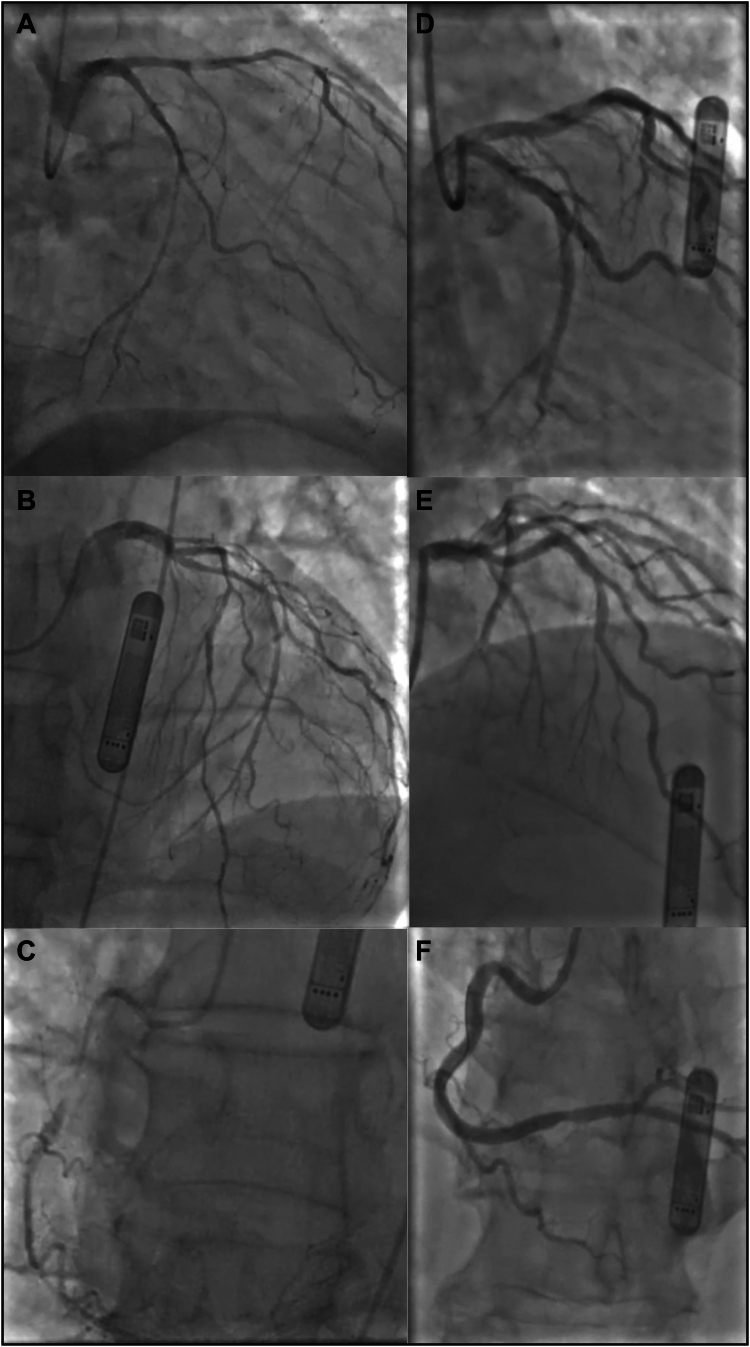
Coronary angiograms. **A:** demonstrates a caudal view of the emergent procedure left coronary and circumflex system, with dramatic improvement and caliber increase in a similar view in **D**. **B:** shows an acute cranial view of the left main and anterior descending artery system, which has multiple non-occlusive stenoses in a small caliber vessel. This contrasts dramatically with (**E)** in the second angiogram, where the arteries have returned to a pre-spasmodic state. **C:** Acute critical stenosis in the mid right coronary artery with TIMI 2 flow, during the emergent angiogram, with resolution and substantial caliber improvement in the second angiogram **(F).** TIMI = thrombolysis in myocardial infarction.

After re-intubation, emergent coronary angiography was performed. Access was attained via the right femoral artery as there was no palpable radial pulse. Given the anterior ECG changes, the left coronary system was first assessed with a 6 Fr Amplatz Left 1 and 6 Fr Q3.5, this revealed small caliber and spastic left anterior descending and circumflex arteries, without occlusion (Figure 3A and 3B). The 450 mcg of intra-coronary glyceryl trinitrate was administered with satisfactory clinical and angiographic response. The right coronary artery was then interrogated with a diagnostic 6 Fr Judkins Right (JR) 4, and spasm was also noted in this vessel, with a high-grade mid-vessel stenosis (Figure 3C). The catheter was swapped out for a 6 Fr JR4 Guide, and when re-engaged moments later, there was further proximal spasm with thrombolysis in myocardial infarction 2 flow and corresponding ST-segment changes. Further nitrate dosing was limited, secondary to hypotension. As a result, percutaneous coronary intervention to the critical lesion with a drug-eluting balloon was undertaken. The ST-segments normalized, and patients went to the intensive care unit (ICU) with a plan for further nitrate, as tolerated, with repeat angiography and optimization when stabilized.

The patient improved in ICU, was extubated and returned for repeat angiography a few days later. There was marked diffuse improvement in caliber of both the left coronary artery and right coronary artery (Figure 3D–3F), in keeping with a mechanism of coronary artery spasm. The patient recovered well, and repeat echocardiogram post-recovery had preserved left ventricular function.

## Discussion

### PF energy

Clinical spasm, and more commonly, non-clinical spasm associated with PF energy is well-described and is considered a class effect. Clinical spasm has been seen when PF is used in close proximity to coronary arteries, with evidence that a distance of <6.5 mm may increase risk.^9^^,^^10^ Pretreatment with nitrates have been shown to reduce the frequency of spasm, with various regimes, including bolus or infusions suggested as potential therapies.^9^^,^^11^ Even with regimented prophylaxis; however, we have seen that spasm is not entirely avoidable.^3^

Notably, PF energy may affect cells within the electric field differently, depending on proximity and pulse dose, sometimes resulting in reversible electroporation if energy thresholds are not met to induce the intended irreversible changes to the cell membrane.^12^ In a similar way, although proximity to the PF catheter results in clinical spasm, effects of extensive PF ablation (PFA) (such as in our case with repeat WACA and PWI) may have more far reaching influence and create subclinical spasm, or reduce the threshold to induce spasm by other factors, such as sympathomimetics or catecholamines.

PF catheters include either single-shot or focal delivery devices, which may use a bipolar or monopolar configuration. There is evidence to suggest impulses from monopolar catheter tips can penetrate deeper into the myocardium, furthermore the catheter used in this case has a wide footprint, which results in greater delivery.^13^ A safety analysis of the multi-national survey on the methods, efficacy, and safety on the post-approval clinical use of pulsed field ablation (MANIFEST-PF) registry of 1568 patients described 1 patient (0.06%) which developed remote diffuse coronary spasm during PVI.^14^ The mechanistic theory was that this phenomenon was sympathetically driven.^15^ Incidence comparison via meta-analyses of thermal sources show that remote diffuse spasm occurred in 0.04% of those treated with cryo-balloon and 0.23% with RF.^16^ In this analysis, they looked at 22,232 patients overall, dwarfing the 1568 numbers in MANIFEST-PF. As PF technologies evolve in delivery modality and configuration, this phenomenon must be closely monitored, especially given the already known association with spasm. Recently, there have been 3 published cases of remote coronary spasm,^6^, ^7^, ^8^ with one of these occurring 45 minutes after the procedure.^8^ Although our case is unique in that all coronary arteries were effected by spasm, this is demonstrating a trend, which must be monitored. However, post-ablation mapping was not performed, and so the extent of the ablated low voltage areas beyond the tagged lesions is unclear.

It is known that spasm is more likely to occur in coronaries with disease and endothelial dysfunction.^17^ Data suggests that up to 11 months after spasm, no changes can be been seen on angiographic follow-up.^18^ Longer-term assessments with endothelial physiology, intravascular imaging, or histopathological follow-up have not been performed.^18^ Preclinical swine studies have suggest that there may be some intimal hyperplasia secondary to PF energy with late luminal narrowing; however, our patient is expected to do well.^19^

### Catheter comparison

The Sphere-9 has preclinical swine models demonstrating the lesion depth, which, like all PFA systems, demonstrates repetition dependency. Four applications of PF demonstrated an acute depth of 8.8 ± 0.74 mm, width of 22.7 ± 2.3 mm, and volume of 2383 ± 548 mm^3^, these are larger than other currently available PFA systems.^20^, ^21^, ^22^, ^23^, ^24^, ^25^ These lesions retracted slightly when other swine were assessed at the 3–4 week mark post-application.

FARAWAVE^TM^ (Boston Scientific), is a common bipolar multi-splined PFA catheter. It was initially assessed in swine models, creating average lesions 6.5 ± 1.7 mm deep by 22.6 ± 4.1 mm wide at 35.5 days.^21^ A further study supported these findings, where they examined healthy vs fibrosed swine myocardium, in different configurations. The average histopathological cross-sectional measurements were found to be 5.6 ± 1.5 mm in focal and 6.6 ± 1.7 mm in basket, respectively.^22^

The FARAFLEX^TM^ large focal catheter (Boston Scientific), can also produce monopolar or bipolar lesions.^23^ Lesion depth in swine were assessed 1 week after ablation. Due to large variation in the results, they were reported with median ± inter-quartile range rather than mean. 4 applications yielded a median depth of 6.5 mm (interquartile range: 5.9–6.9 mm) in monopolar, and of note 2 lesions had depth >11 to 12 mm. In bipolar, median depth was 5.5 mm (interquartile range: 5.1–6.8 mm) with a maximum depth of <8 mm. Median lesion width was 20.6 mm (range: 11.5–41.6) monopolar and 21.9 mm (range: 13.1–39.8) bipolar.

The VARIPULSE^TM^ variable loop catheter (Johnson & Johnson MedTech) was assessed in swine.^24^ Repetitive applications and increased force generated deeper lesions. The greatest mean depths were achieved with 6 applications and 30 g of force, 7.3 ± 0.9 mm. Six applications with 15 g of force attained 6.5 ± 0.7 mm.

## Anesthetic

The process of tracheal extubation results in increased patient sympathetic activity, this may increase the risk of vasospasm.^25^ ST-elevation associated with extubation has been described; however, this is exceedingly rare, limited to case reports despite this process having occurred an immeasurable number of times worldwide.^26^^,^^27^ Endogenous catecholamine-mediated coronary vasoconstriction is very limited in normal coronary arteries.^17^ Intravascular ultrasound of our patients coronary arteries on his repeat angiogram only demonstrated mild disease, leading us to suspect further contributing factors.

As noted, the patient did receive phenylephrine. This medication activates alpha-1 receptors resulting in vasoconstriction and increased systemic blood pressure. Alpha-1 receptors are also present in the epicardial coronary arteries.^28^ Phenylephrine was not administered after the start of the case until the post-ROSC phase. With its widespread use and an effective half-life of just 5 minutes, contribution of this medication to the clinical picture is unlikely.^29^

Protamine sulfate is the reversal agent for unfractionated heparin in a 1:1 unit ratio.^30^ It is given in many centers post-PVI, with evidence suggesting that it minimizes vascular complications without increasing thrombotic events.^31^ It may cause anaphylaxis, and as a consequence there have been rare reports of coronary vasospasm (Kounis syndrome) with its use, including post-PVI.^32^, ^33^, ^34^ Slow infusions, peripheral administration, or a “test dose” earlier in the case may allow for earlier detection of potential hemodynamic conpromise.^30^ We slowly administered 50 mg (a single 5 mL vial, with the equivalent of 7000 anti-heparin units), which is the maximum bolus dose.^35^ As the spasm occurred within 30 minutes of the administration, this is a potential explanation. Unfortunately, as a consequence of the cardiac arrest management and the need for emergent stabilization, coronary angiography, and intervention, a timely tryptase level was not attained to categorically include or exclude this diagnosis.

Doxapram, an alkaloid used to treat respiratory and central nervous system depression secondary to general anesthesia, was administered at the time of extubation. The primary medication site of action is uncertain, but believed to be the brainstem respiratory center or the peripheral aortic/carotid chemoreceptors.^36^ This medication has been shown to have a mild vasoactive pressor effect, theorized via mediation of catecholamine release. Side effects include hypertension, flushing, sweating, and muscle spasticity.^37^ It is reported to potentiate the effect of vasoactive medication; however, doxapram has never been reported to cause coronary artery spasm alone.

Sugammadex is a modified y-cyclodextrin, and is very widely used to reverse the effects of aminosteroid neuromuscular paralytic agent, rocuronium.^38^ This medication is considered very safe. There have been case reports of sugammadex-associated coronary spasm, with mechanisms of either primary spasm or secondary to anaphylaxis (Kounis syndrome).^39^, ^40^, ^41^, ^42^, ^43^ One case occurred after RF ablation of atrial fibrillation and atrial flutter.^43^ That case also included spasm of the right coronary artery, as it is the most susceptible to spasm, and the event was reversed by nitrates and calcium channel blockers. Similar to protamine, given the timing of the episode shortly after administration, this medication could certainly have played a role.

## Conclusion

This was a highly atypical case of coronary artery spasm after a PFA causing ST-elevation and cardiac arrest. The case was complicated by the limited ability to use nitrates due to acute hypotension post-ROSC. Areas of clinical coronary spasm associated with PFA are usually anatomically adjacent to delivery; however, this case had diffused spasm across the left and right coronary artery territories, suggesting a systemic driver. As the spasm occurred following extubation (which is associated with catecholamine release) and administration of medication, such as protamine, doxapram and sugammadex, this is suspected as the primary mechanism via sympathetic activation or Kounis syndrome. However, such a phenomenon is exceedingly rare, despite widespread use of these medicines worldwide.

Because PF is known to also cause subclinical spasm, and recent cases describe remote coronary spasm in space or time post-PVI, it raises questions as to whether extensive ablation with a powerful monopolar catheter could contribute to this phenomenon, potentially by reducing the threshold for sympathetic diffuse distal clinical spasm. Given the extreme nature of this complication and cardiac arrest, more research is needed into the concurrent use of vasoactive medications, protamine, or sugammadex with PF energy.

## Disclosures

The authors have no conflicts of interest to disclose.

## References

[1] Gunawardene M.A., Schaeffer B.N., Jularic M. (2021). Coronary spasm during pulsed field ablation of the mitral isthmus line. JACC Clin Electrophysiol.

[2] Monaco C., Menè R., Yokoyama M. (2024). Coronary vasospasm during pulse-field focal ablation of the cavotricuspid isthmus observed with intravascular ultrasound. JACC Clin Electrophysiol.

[3] Gunawardene M.A., Hartmann J., Tigges E., Jezuit J., Willems S. (2024). Word of caution: clinically apparent coronary spasm following pulsed field cavotricuspid isthmus ablation despite nitroglycerin prophylaxis - a case report. Eur Heart J Case Rep.

[4] Husted S., Nedergaard O.A. (1981). Inhibition of adrenergic neuroeffector transmission in rabbit pulmonary artery and aorta by adenosine and adenine nucleotides. Acta Pharmacol Toxicol.

[5] Jackson W.F. (2021). Calcium-dependent ion channels and the regulation of arteriolar myogenic tone. Front Physiol.

[6] Becker B.D., Francois C., Smet M.D. (2023). Severe coronary spasm occurring remotely from pulsed field application during right inferior pulmonary vein isolation. J Interv Card Electrophysiol.

[7] Schaack D., Plank K., Bordignon S. (2024). Severe ST-segment elevation and AV block during pulsed-field ablation due to vasospastic angina — a novel observation. J Interv Card Electrophysiol.

[8] Luther V., Chiong J., James C., Modi S., Gupta D., Hung J. (2025). Diffuse right coronary artery spasm occurring forty-five minutes post pulsed field ablation for atrial fibrillation. Heart Rhythm.

[9] Reddy V.Y., Petru J., Funasako M. (2022). Coronary arterial spasm during pulsed field ablation to treat atrial fibrillation. Circulation.

[10] Higuchi S., Buck E.D., Schneider C.W., Gerstenfeld E.P. (2023). What is a safe distance for delivering pulsed field ablation near coronary arteries?. Heart Rhythm.

[11] Malyshev Y., Neuzil P., Petru J. (2024). Nitroglycerin to ameliorate coronary artery spasm during focal pulsed-field ablation for atrial fibrillation. JACC Clin Electrophysiol.

[12] Mannion J., Rathore F., David J., Lyne J. (2025). Using a novel pulsed field ablation technique to identify the critical isthmus within a tachycardia circuit. J Innov Card Rhythm Manag.

[13] Ryul K., Chun J., Miklavčič D. (2024). State-of-the-art pulsed field ablation for cardiac arrhythmias: ongoing evolution and future perspective. Europace.

[14] Turagam M.K., Neuzil P., Schmidt B. (2023). Safety and effectiveness of pulsed field ablation to treat atrial fibrillation: one-year outcomes from the MANIFEST-PF registry. Circulation.

[15] Gehl J., Skovsgaard T., Mir L.M. (2002). Vascular reactions to in vivo electroporation: characterization and consequences for drug and gene delivery. Biochim Biophys Acta.

[16] Nakamura T., Takami M., Fukuzawa K. (2021). Incidence and characteristics of coronary artery spasms related to atrial fibrillation ablation procedures - large-scale multicenter analysis. Circ J.

[17] Baumgart D., Haude M., Günter G. (1999). Augmented α-adrenergic constriction of atherosclerotic human coronary arteries. Circulation.

[18] Malyshev Y., Neuzil P., Petru J. (2024). Does acute coronary spasm from pulsed field ablation translate into chronic coronary arterial lesions?. JACC Clin Electrophysiol.

[19] Higuchi S., Im S.I., Stillson C. (2022). Effect of epicardial pulsed field ablation directly on coronary arteries. JACC Clin Electrophysiol.

[20] Yavin H.D., Higuchi K., Sroubek J., Younis A., Zilberman I., Anter E. (2021). Pulsed-field ablation in ventricular myocardium using a focal catheter. Circ Arrhythm Electrophysiol.

[21] Koruth J.S., Kuroki K., Iwasawa J. (2019). Endocardial ventricular pulsed field ablation: a proof-of-concept preclinical evaluation. Europace.

[22] Im S.I., Higuchi S., Lee A. (2022). Pulsed field ablation of left ventricular myocardium in a swine infarct model. JACC Clin Electrophysiol.

[23] Kueffer T., Casoni D., Goepfert C. (2025). Dose-dependent ventricular lesion formation using a novel large-area pulsed field ablation catheter: a preclinical feasibility study. Heart Rhythm.

[24] Biase L.D., Marazzato J., Gomez T. (2024). Application repetition and electrode-tissue-contact results in deeper lesions using a pulsed-field ablation circular variable loop catheter. Europace.

[25] Nishina K., Mikawa K., Maekawa N., Obara H. (1995). Attenuation of cardiovascular responses to tracheal extubation with diltiazem. Anesth Analg.

[26] Lindsay P.J., Frank R.C., Bittner E.A., Berg S., Chang M.G. (2020). ST elevations and ventricular tachycardia secondary to coronary vasospasm upon extubation. Case Rep Anesthesiol.

[27] Akata T., Hoka S., Takahashi S., al (1992). Coronary artery spasm immediately following extubation of the trachea. J Anesth.

[28] Jensen B.C., Swigart P.M., Laden M.E., DeMarco T., Hoopes C., Simpson P.C. (2009). The Alpha-1D is the predominant Alpha-1-adrenergic receptor subtype in human epicardial coronary arteries. J Am Coll Cardiol.

[29] Phenylephrine: full prescribing information US Food and Drug Administration. https://www.accessdata.fda.gov/drugsatfda_docs/label/2012/203826s000lbl.pdf.

[30] Applefield D., Krishnan S. (2020). Protamine. Treasure Island, FL: StatPearls Publishing. https://www.ncbi.nlm.nih.gov/books/NBK547753/.

[31] Gurses K.M., Kocyigit D., Yalcin M.U. (2015). Safety and efficacy outcomes of protamine administration for heparin reversal following cryoballoon-based pulmonary vein isolation. J Interv Card Electrophysiol Int J Arrhythmias Pacing.

[32] Itoh T., Kanaya Y., Komuro K. (2022). Kounis syndrome caused by protamine shock after coronary intervention: a case report. J Cardiol Cases.

[33] Amro M., Mansoor K., Amro A., Okoro K., Okhumale P.I. (2020). Kounis syndrome induced by protamine sulfate. Cureus.

[34] Lee S., Nikai T., Kanata K., Koshizaki M., Nomura T., Saito Y. (2005). A case of severe coronary artery spasm associated with anaphylactic reaction caused by protamine administration. Masui Jpn J Anesthesiol.

[35] Protamine sulphate Leo Pharma 1400 anti-heparin IU/ml solution for injection and infusion. Medicines.ie. https://www.medicines.ie/medicines/protamine-sulphate-leo-pharma-1400-anti-heparin-iu-ml-solution-for-injection-and-infusion-33466/spc#spc.

[36] Yost C.S. (2006). A new look at the respiratory stimulant doxapram. CNS Drug Rev.

[37] Health products regulatory authority summary of product characteristics: Doxapram hydrochloride 2mg/ml solution for Infusion. Health Products Regulatory Authority (HPRA). https://assets.hpra.ie/products/Human/22387/Licence_PA0857-003-002_05102022104052.pdf.

[38] Fuchs-Buder T., Meistelman C., Raft J. (2013). Sugammadex: clinical development and practical use. Korean J Anesthesiol.

[39] Ko M.J., Kim Y.H., Kang E., Lee B.C., Lee S., Jung J.W. (2016). Cardiac arrest after sugammadex administration in a patient with variant angina: a case report. Korean J Anesthesiol.

[40] Hoshino K., Kato R., Nagasawa S., Kozu M., Morimoto Y. (2015). A Case of repetitive cardiac arrest due to coronary vasospasm after sugammadex administration. Masui.

[41] Okuno A., Matsuki Y., Tabata M., Shigemi K. (2018). A suspected case of coronary vasospasm induced by anaphylactic shock caused by rocuronium-sugammadex complex. J Clin Anesth.

[42] Yanai M., Ariyoshi K. (2020). Two cardiac arrests that occurred after the administration of sugammadex: a case of Kounis syndrome. Case Rep Emerg Med.

[43] Boo K.Y., Park S.H., Park S.K., Na C., Kim H.J. (2023). Cardiac arrest due to coronary vasospasm after sugammadex administration -a case report. Korean J Anesthesiol.

